# Supplementary material for: Iron ions regulate antifungal HSAF biosynthesis in Lysobacter enzymogenes by manipulating the DNA-binding affinity of the ferric uptake regulator (Fur)
Source: Microbiol Spectr. 2023 Sep 22;11(5):e00617-23. doi: 10.1128/spectrum.00617-23 (PMC10581043; doi:10.1128/spectrum.00617-23)
Supplement: Fig. S1-S3, Table S1 — RT-qPCR validation, OD600 and HSAF production, PCR and SDS-PAGE, and Primers for RT-qPCR. [file spectrum.00617-23-s0001.docx]

**Supplementary materials**

**Iron ions regulate antifungal HSAF biosynthesis in *Lysobacter enzymogenes* by manipulating the DNA-binding affinity of the ferric uptake regulator (Fur)**

**Bao Tang ^a,b,1^ , Bo Wang ^a,1^, Zhizhou Xu ^a,c^, Rouxian Hou ^a,c^, Min Zhang ^a^, Xian Chen ^a^, Youzhou Liu ^a^, Fengquan Liu ^a,d^*****

^a^ Institute of Plant Protection, Jiangsu Key Laboratory for Food Quality and Safety-State Key Laboratory Cultivation Base of Ministry of Science and Technology, Jiangsu Academy of Agricultural Sciences, Nanjing 210014, China.

^b^ School of Life Sciences, Jiangsu University, Zhengjiang 212013, China;

^c^ College of Plant Protection, Nanjing Agricultural University, Nanjing 210095 China;

^d^ College of Plant Protection, Hainan University, Haikou 570228, China.

^1^ These authors had equal contributions to this work.

* Corresponding author

Fengquan Liu; Institute of Plant Protection, Jiangsu Academy of Agricultural Sciences, Nanjing 210014, China; Tel/Fax: +86-25-84390873;

E-mail address: fqliu20011@sina.com (F. Liu).


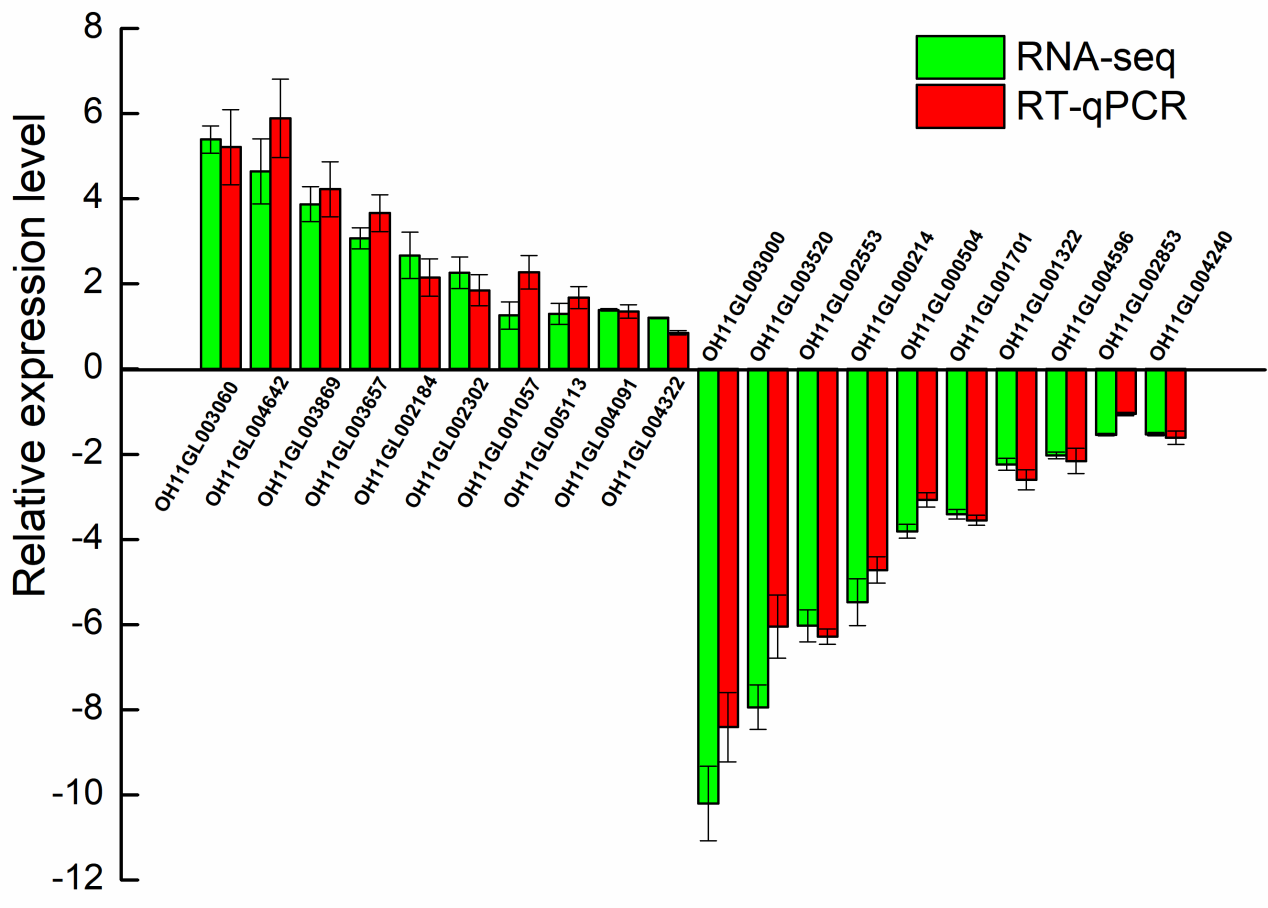


**FIG. S1** RT-qPCR validation of RNA-seq data. The X axis shows the selected genes, and the Y axis shows the relative expression level of each gene.


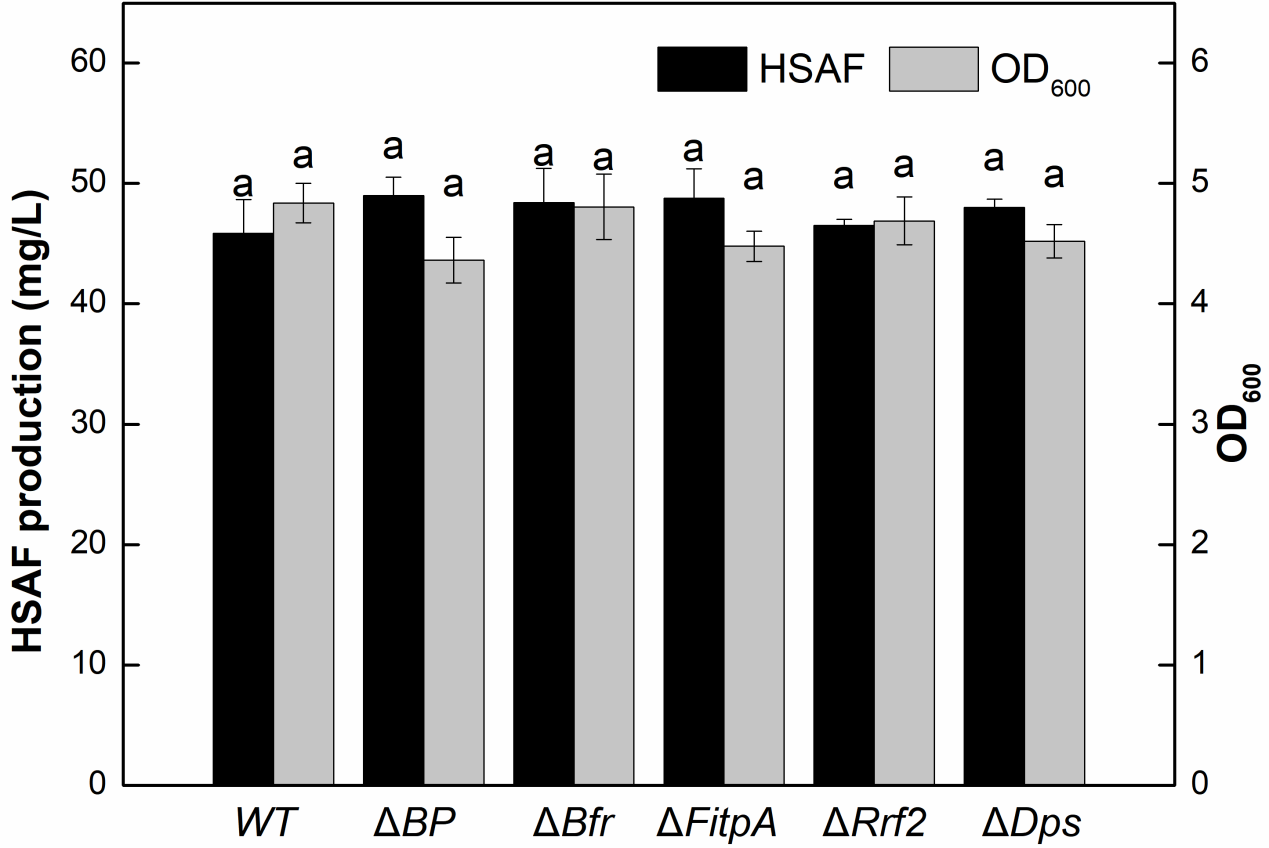


**FIG. S2** OD_600_ and HSAF production in wild-type and mutant strain cultured in the IDM. Values represent the means ± SD. Significant differences calculated by Tukey's LSD, p < 0.05. Small letters correspond to significance values of different treatment.


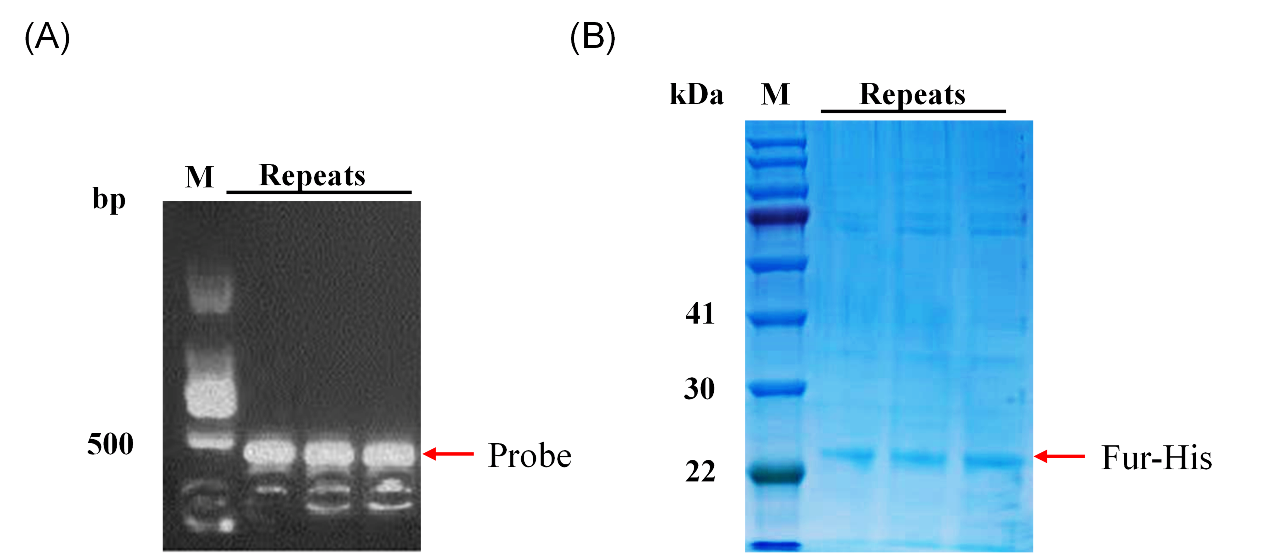


**FIG. S3** (A) PCR result of P_HSAF_; (B) SDS-PAGE analysis of purified His-tagged Fur.

**Table S1** Primers for RT-qPCR verification

| **Primer names** | **Sequence (5’→3’)** |
| --- | --- |
| RT-*3060*-up | TGCTGATCGCGCTGCTGTGGTT |
| RT-*3060-*do | ATAACGGCCGCGCTCGATCT |
| RT-*4642*-up | ATGACCTCCCTGGACACCTC |
| RT-*4642-*do | GATCTTGTTCGCGGTCTCGG |
| RT-*3869*-up | ACAGGCGAGTATGAGGGGCA |
| RT-*3869-*do | GACACCTGACGTAAACCGCG |
| RT-*3657*-up | TCGACGAAGCCCTGCACGAA |
| RT-*3657-*do | CGCCCCACATATCGAGGAAC |
| RT-*2184*-up | GTCGCCGATCACCACGATCA |
| RT-*2184-*do | ACAGCGTGCCGATGTCCTTG |
| RT-*2302*-up | GTGCGCTTCACCGAGTCTGA |
| RT-*2302-*do | TAGTTGTAGTCGGCGCAGCC |
| RT-*1057*-up | CGGTGAGCTGCAAGGAGTGGAA |
| RT-*1057-*do | TGCACGCGGTTGAACCAGAC |
| RT-*5113*-up | GCTCCGCAAGACGCCCTTAA |
| RT-*5113-*do | TGGAGTCCGTCATGGTCGAT |
| RT-*4091*-up | CGACGAATGGGCGGTGGATT |
| RT-*4091-*do | GGCGCGGGTTTCGTAATCGA |
| RT-*4322*-up | ATGGCCAAGAAAACCGCTTC |
| RT-*4322-*do | GCCAGTAATCGCGCAGGAAC |
| RT-*3000*-up | AGCGGCGACGTCAACGAACT |
| RT-*3000-*do | TTCGTTGTCCATGCGCTCGC |
| RT-*3520*-up | CGCATGAAGATCGACGCCAA |
| RT-*3520-*do | GGTTCTTGTCGAGCTCGTCG |
| RT-*2553*-up | GTCCACTTCACCGTCGCCTT |
| RT-*2553-*do | TTCTCGTGCAGGTGGAAGGC |
| RT-*0214*-up | AAAAACACTTTCTCGCAAGC |
| RT-*0214-*do | TGAGTTCCTTGTCGAAGCAG |
| RT-*0504*-up | ATGGCGATGAACACCGACCC |
| RT-*0504-*do | GTGCAGATCGCGCAGAGCCG |
| RT-*1701*-up | AGCTCCTGCCAGCACGAACA |
| RT-*1701-*do | GGTCTTGTCGTCGGACATGC |
| RT-*1322*-up | TGTGGATAGTCCAATACGCC |
| RT-*1322-*do | GATATCGGTCGGCATCTTGC |
| RT-*4596*-up | CTTTCGTCGGCACCATGCAG |
| RT-*4596-*do | CGTTGAAGGCCTTGAGCAGG |
| RT-*2853*-up | GCCGCTGACCTTGCTGTACT |
| RT-*2853-*do | ACGCACCTGAAAATCCGCAT |
| RT-*4240*-up | ATGACGGAACGCACGACGAA |
| RT-*4240-*do | ACCACGTCGCCCTGGAAATC |
| RT-16S-up | ACATTTGATGAACGTCGGCG |
| RT-16S-do | CCACTTTCACCCGTAGGTCG |
